# Biological risk factors for suicidal behaviors: a meta-analysis

**DOI:** 10.1038/tp.2016.165

**Published:** 2016-09-13

**Authors:** B P Chang, J C Franklin, J D Ribeiro, K R Fox, K H Bentley, E M Kleiman, M K Nock

**Affiliations:** 1 Department of Emergency Medicine, Columbia University Medical Center, New York, NY, USA; 2 Department of Psychology, Vanderbilt University, Nashville, TN, USA; 3 Department of Psychology, Harvard University, Cambridge, MA, USA; 4 Department of Psychology, Boston University, Boston, MA, USA

## Abstract

Prior studies have proposed a wide range of potential biological risk factors for future suicidal behaviors. Although strong evidence exists for biological correlates of suicidal behaviors, it remains unclear if these correlates are also risk factors for suicidal behaviors. We performed a meta-analysis to integrate the existing literature on biological risk factors for suicidal behaviors and to determine their statistical significance. We conducted a systematic search of PubMed, PsycInfo and Google Scholar for studies that used a biological factor to predict either suicide attempt or death by suicide. Inclusion criteria included studies with at least one longitudinal analysis using a biological factor to predict either of these outcomes in any population through 2015. From an initial screen of 2541 studies we identified 94 cases. Random effects models were used for both meta-analyses and meta-regression. The combined effect of biological factors produced statistically significant but relatively weak prediction of suicide attempts (weighted mean odds ratio (wOR)=1.41; CI: 1.09–1.81) and suicide death (wOR=1.28; CI: 1.13–1.45). After accounting for publication bias, prediction was nonsignificant for both suicide attempts and suicide death. Only two factors remained significant after accounting for publication bias—cytokines (wOR=2.87; CI: 1.40–5.93) and low levels of fish oil nutrients (wOR=1.09; CI: 1.01–1.19). Our meta-analysis revealed that currently known biological factors are weak predictors of future suicidal behaviors. This conclusion should be interpreted within the context of the limitations of the existing literature, including long follow-up intervals and a lack of tests of interactions with other risk factors. Future studies addressing these limitations may more effectively test for potential biological risk factors.

## Introduction

Suicide is a pressing public health problem. Each year there are an estimated one million suicide deaths worldwide in addition to ~25 million non-lethal suicide attempts.^1, 2, 3^ Despite a large increase in suicide research over the last five decades, there has been no appreciable decline in the rates of suicidal behaviors.^4^ This lack of progress highlights the need for a new suicide research strategy.^5^

The study of potential biomarkers may aid researchers and clinicians in the prediction of future suicidal behaviors as well as help shed light on the etiology and underlying mechanisms associated with suicidal behavior. A number of biological factors have been proposed as possible risk factors for suicidal behaviors.^6, 7, 8^ However, most studies in this area are cross-sectional, making it unclear which biological factors are correlates (that is, factors that co-occur with suicidal behaviors) and which are risk factors (that is, factors that predict future suicidal behaviors). ^9^ Furthermore, drawing conclusions based on the existing literature is complicated by the presence of conflicting findings from existing longitudinal studies of biological factors. For example, some studies have shown that nonsuppression during the dexamethasone suppression test predicts future suicidal behaviors (for example, odds ratios (ORs)>10),^10, 11^ whereas others report that dexamethasone suppression test nonsuppression predicts significantly fewer suicidal behaviors.^12^

To resolve such discrepancies and summarize current knowledge about biological risk factors for suicidal behaviors, we conducted a meta-analysis of longitudinal studies that have tested whether biological factors predict suicidal behaviors. We had four specific aims. First, we examined the descriptive characteristics of this literature, including the number of studies, frequency of studies across time, population types and follow-up lengths. Second, we investigated whether any biological factors qualified as statistically significant risk (or protective) factors and, if so, we estimated the magnitude of these factors. Third, we examined publication bias within this literature. Fourth, we tested study population type and follow-up length as moderators of risk factor magnitude.

## Materials and methods

Our meta-analysis and systematic review was conducted in accordance with the Meta-analysis of Observational Studies in Epidemiology^13^ guidelines and the Preferred Reporting Items for Systematic Reviews and Meta-analyses^14^ standard.

### Data sources, study selection and eligibility criteria

Studies were identified through systematic literature searches conducted through 1 January 2015 using PubMed, Psycinfo and Google Scholar. Search terms included variants of the words cludeitudinal words ‘longitudinal' (for example, longitudinally, predicts, prediction, prospective, prospectively, future, later, follow-up) ‘suicide' (for example, suicide, suicidal behavior, suicide attempt, suicide death, suicide plan, suicide thoughts, suicide ideation, suicide gesture, suicide threat, suicidality, self-poisoning, deliberate self-harm, DSH, self-injury, self-harm, self-mutilation, self-cutting, cutting, self-burning, nonsuicidal self-injury, NSSI). This generated a total of unique 2541 publications. We conducted this broad suicide-related search because some studies that include biological predictors of suicidal behaviors do not include biological-related terms in the keywords or abstracts.

**Figure 1:**
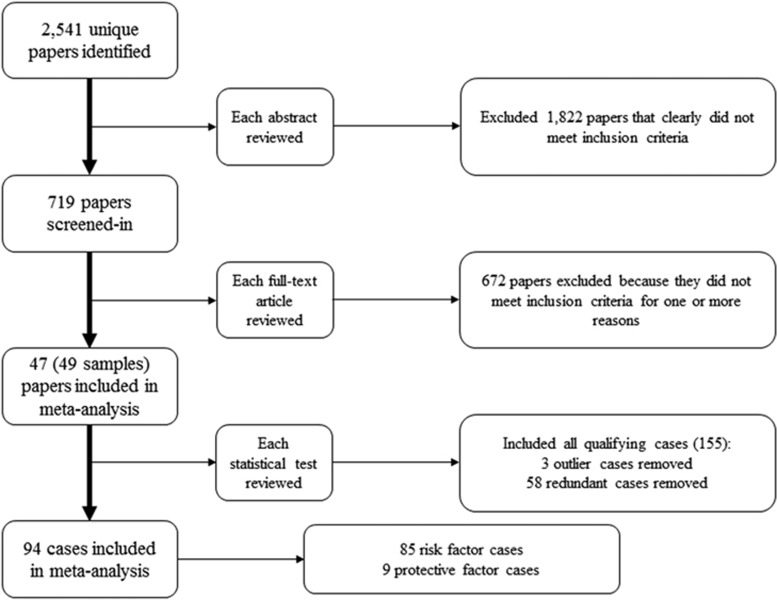
PRISMA diagram for the present meta-analysis.

Eligible studies were required to report at least one longitudinal analysis using a biological factor to predict suicidal behaviors in any population, year or location. Although genetic studies typically did not include longitudinal designs, they qualified because they inherently included longitudinal associations (that is, genes were present before suicidal behaviors). We first screened the abstracts of all 2541 articles to examine whether they longitudinally predicted a specific suicide-related outcome (that is, suicide ideation, plan, gesture, attempt or death). A total of 719 articles were retained after this screening. Remaining articles were read in full to determine whether they included a biological predictor of suicidal behaviors and whether statistical information was sufficient for meta-analysis. Forty-seven studies (comprising 49 samples) met these criteria, producing 155 potential prediction cases (that is, instances where a biological variable was used to longitudinally predict a suicide-related outcome; Figure 1).^10, 15, 16, 17, 18, 19, 20, 21, 22, 23, 24, 25, 26, 27, 28, 29, 30, 31, 32, 33, 34, 35, 36, 37, 38^

Prediction cases were examined for redundancy in order to ensure case independence. Data redundancy occurred when: (1) multiple publications appeared to report the same or overlapping data (*n*=3) or (2) a single publication tested multiple levels of a particular predictor (for example, quartiles of forced vital capacity; *n*=55).^15^ In these instances, we included prediction cases that were most inclusive (publication with largest sample), most extreme comparisons (for example, lowest and highest quartiles) or produced the strongest effect. Cases greater than three s.d. away from the mean were considered outliers and omitted from analyses (*n*=3, ORs>22);^10, 39^ however, we note that the results of our meta-analysis were virtually identical when these cases were included. After these exclusions, a total of 94 prediction cases were included in this meta-analysis.

### Data extraction

In addition to the predictor, outcome and statistics relevant to the longitudinal analyses of interest, the following data were extracted from each study: authors; publication year; follow-up length; number of participants with a suicide-related outcome; and sample type (that is, general population, clinical and suicidal). We examined the number of participants with a suicide-related outcome rather than overall study sample size for two reasons: (1) overall sample sizes varied widely (range=15 to 846,907) and (2) reliability of effect size estimates was much more closely related to the number of participants with suicide-related outcomes than the overall sample size. With regard to sample type, samples were defined as ‘self-injurious' if they included at least one participant selected for prior suicidal thoughts or behaviors and ‘clinical' if they did not meet the ‘suicidal' criterion but did include at least one participant selected for psychopathological symptoms. Samples were defined as ‘general population' if they did not meet either of these two criteria. Category and subcategory codes were assigned to each predictor. These categories reflected separate biological systems, metabolites, processes, or tests. All authors reached agreement on all category and subcategory designations.

### Statistical analyses

Comprehensive Meta-Analysis, Version 3.0 (Biostat, Englewood, NJ, USA) software was used to conduct meta-analyses.^40^ Unadjusted estimates were used when available (*n*=84; adjustment status of prediction cases did not moderate any findings). Correlations, independent group means, and 2 × 2 contingency tables were converted into OR estimates if ORs were not available. OR; *n*=75 and hazard ratio (HR; *n*=19) estimates were analyzed independently because HRs cannot be converted into ORs. Between-study heterogeneity, which was quantified using *I*^2^ tests, was expected to be high. As such, all meta-analyses used random effects models to account for this heterogeneity. To evaluate the effects of continuous moderators, we employed meta-regression using a random effects model with unrestricted maximum likelihood.

To evaluate publication bias, we examined funnel plots and calculated Duval and Tweedie's Trim and Fill tests. Funnel plots chart standardized effect sizes against variance around the observed meta-analytic mean. Compared with large studies, small studies are more likely to obtain extremely positive and extremely negative results. Owing to publication bias toward publishing positive findings, publication bias tends to produce large positive effects from smaller studies. This can be visually examined within funnel plots. In the absence of publication bias, the funnel plot is symmetrical (and resembles an inverted funnel), with studies equally likely to fall above and below the mean regardless of study size. Publication bias produces asymmetry in this plot, as small studies with positive findings (especially large positive findings) are more likely to be published than small studies with negative findings Duval and Tweedie's Trim and Fill test helps to quantify and account for publication bias observed in funnel plots. This test determines how many studies are missing within a funnel plot, imputes effect sizes for these missing studies, and calculates what the meta-analytic effect size would have been had these studies been included.

### Data analytic plan

We first calculated the descriptive characteristics of this literature, including number of prediction cases across time, outcome and sample type, as well as follow-up lengths and the number of participants with a suicide-related outcome. Second, we analyzed the ability of biological risk factors as a whole (that is, without dividing factors into categories) to predict suicide attempt and suicide death, and accounted for publication bias within these analyses. Third, we examined the ability of specific categories of biological risk factors to predict suicide attempt and suicide death, and accounted for publication bias. The size of the cerebrospinal fluid (CSF) metabolite category allowed for additional analyses of sub-categories related to specific metabolites. Fourth, we investigated the ability of protective biological factors (that is, factors that would be hypothesized *a priori* to be associated with less suicidal behavior) to reduce the likelihood of suicide attempt and suicide death.

## Results

### Descriptive characteristics of the literature

Our review of the literature found biological factors predicting suicidal behavior or death by suicide across several categories (Table 1). Included studies were published between 1976 and 2014, with the number of prediction cases increasing between pre-1985 (*n*=5), to 1985–1994 (*n*=10), to 1995–2004 (*n*=37), and then leveling off during 2005–2014 (*n*=42). Prediction cases primarily included either suicide attempt (*n*=35, 37.23%) or suicide death as outcomes (*n*=58, 61.70%); only one case was used to predict ideation (not sufficient for meta-analysis), and none included suicide plans or gestures as an outcome. A total of 9 cases (9.57%) were classified as protective factors (that is, factors hypothesized to decrease the likelihood of a negative outcome among those at risk such as omega 3 fatty acids) rather than risk factors; these cases were all used to predict suicide death.^15, 16, 17^ In subsequent sections, we analyzed these cases separately from risk factors. Across all outcomes and factor types, general population samples were most common (*n*=41; 43.61%), followed by clinical (*n*=26; 27.66%) and suicidal samples (*n*=27; 28.72%).

Prediction cases typically included a small number of participants with suicide-related outcomes, with a median of 21 such participants (*M*=88.45; s.d.=173.13; range=2–800). Long follow-ups were common, with a mean follow-up length of 109.99 months (s.d.=117.41; *Mdn*=60; range=6–480) across outcomes. Over 50% of prediction cases had follow-up intervals of 5 years or longer, and only two cases had follow-ups of <1 year.^18, 19^

### Overall risk analyses

#### Suicide attempts

**Figure 2:**
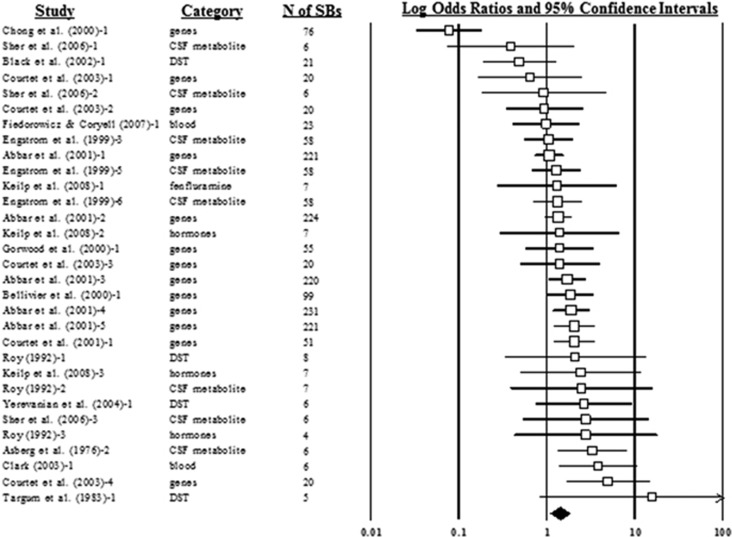
Forest plot for suicide attempt outcome cases (weighted mean odds ratio and risk factor cases). CSF, cerebrospinal fluid; *N* of SBs, number of participants who engaged in suicidal behaviors.

**Figure 3:**
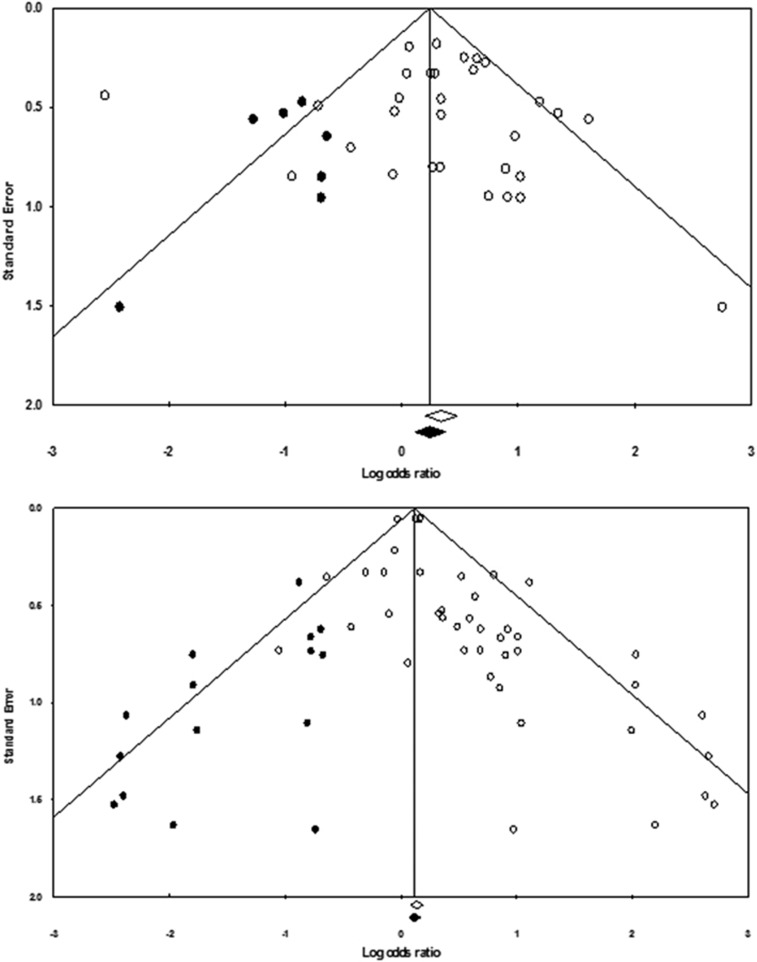
Funnel plots for weighted mean odds ratio analyses for suicide attempt (top) and suicide death (bottom) outcomes. White circles represent observed cases, black circles represent cases imputed by Duval and Tweedie analyses to account for publication bias.

The overall effect of biological factors predicting suicide attempts produced a weighted mean OR of 1.41 (95% CI: 1.09–1.81; Figure 2), with moderate heterogeneity across cases (*n*=31 cases; *I*^2^=62.16%). Consistent with the appearance of an asymmetrical funnel plot (Figure 3), publication bias analyses estimated that seven cases were missing below the mean. Inclusion of these missing cases would reduce the overall effect to a weighted mean OR of 1.17 (0.91–1.51) and render it nonsignificant. HR analyses only included four prediction cases (all from the same study), but yielded similar results, with a weighted mean HR of 1.01 (0.99–1.03). Publication bias analyses revealed that one HR case was missing and that, if it had been included, the weighted mean HR would have been 1.00 (0.98–1.03).

#### Suicide death

**Figure 4:**
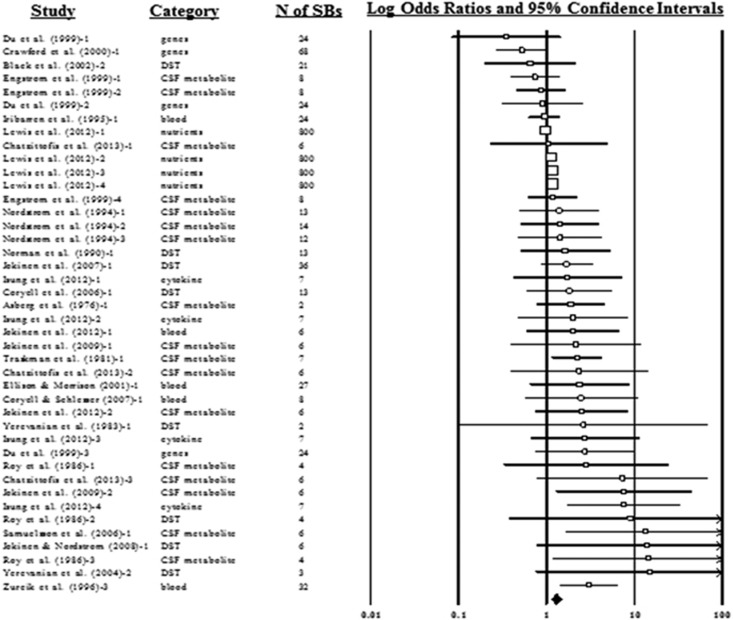
Forest plot for suicide death outcome cases (weight mean odds ratio and risk factor cases only). CSF, cerebrospinal fluid; *N* of SBs, number of participants who engaged in suicidal behaviors.

Biological factors predicting suicide death generated a weighted mean OR of 1.28 (1.13–1.45; Figure 4). There was moderate heterogeneity across cases (*n*=42; *I*^2^=45.45%). As with suicide attempts, there was an asymmetrical funnel plot (Figure 3). Publication bias analyses estimated that there were 15 missing cases below the mean and that, if included, these cases would reduce the mean weighted OR to 1.13 (0.99–1.32). HR analyses included only seven cases from two studies and produced a weighted HR of 1.38 (1.02–1.86), with moderate heterogeneity (*I*^2^=68.49).

### Risk factor category analyses

#### Blood-related factors

There were few relevant prediction cases for either attempt (*n*=2; weighted mean OR=1.89 (0.50–7.23)) or death outcomes (*n*=5; weighted mean OR=1.82 50 (0.99–3.34)), and neither analysis produced a significant effect.

#### Cerebrospinal fluid metabolites

A total of eight prediction cases included suicide attempt as an outcome and 17 included suicide death as an outcome. For attempt, the weighted mean OR was 1.41 (0.81–2.45). The weighted mean OR for death was 1.65 (1.19–2.30), but publication bias analyses estimated that seven cases below the mean were missing; inclusion of these cases would have reduced the weighted mean OR to 1.29 (0.90–1.84).

Suicide attempt subcategory analyses on specific CSF metabolites did not reveal any significant effects, with small and highly variable effect sizes across corticotrophin-releasing hormone (*n*=1; OR=2.49 (0.39–16.14)), dopamine (*n*=2; weighted mean OR=1.23 (0.67–2.26)), norepinephrine (*n*=2; weighted mean OR=1.47 (0.80–2.69)) and serotonin metabolites (*n*=3; weighted mean OR=1.27 (0.46–3.54)). Suicide death subcategory analyses revealed nonsignificant effects for metabolites of cortisol (*n*=1; weighted mean OR=2.35 (0.38–14.47)), DHEAS (*n*=1; OR=1.06 (0.22–5.08)), dopamine (*n*=4; weighted mean OR=1.49 (0.60–3.70)), norepinephrine (*n*=2; weighted mean OR=0.99 (0.56–1.73)), and oxytocin (*n*=1; weighted mean OR=2.52 (0.74–8.57)). The effect of the CSF serotonin metabolite (*n*=8) was significant with a weighted mean OR of 2.15 (1.34–3.44). Publication bias analyses indicated that three serotonin metabolite cases below the mean were missing, and estimated an adjusted weighted mean OR of 1.69 (0.97–2.92).

#### Cytokines

Isung *et al.* (2012) was the only study included in the meta-analysis that examined cytokines. This study examined several variants of interleukin; to reduce dependence, we only included the strongest prediction case among these variants. In total, we analyzed four cytokine prediction cases for suicide death, producing a significant weighted mean OR of 2.87 (1.38–6.00). No publication bias was detected for this analysis; however, caution should be exercised when interpreting these findings because (a) all cases came from one study and (b) we only included in analyses the strongest of seven interleukin effects. No cytokine prediction cases included a suicide attempt outcome.

#### Dexamethasone suppression test

Four prediction cases used dexamethasone suppression test to predict suicide attempt. These produced a nonsignificant weighted mean OR of 1.49 (0.58–3.82). Eight prediction cases included suicide death as an outcome and generated a significant weighted mean OR of 1.75 (1.05–2.90). Publication bias analyses indicated that there were three prediction cases missing below the mean. If these had been included, the weighted mean OR would have been nonsignificant (OR=1.45; 0.78–2.68)

#### Fenfluramine

There was only one case for this category (predicting suicide attempt), precluding any meta-analytic calculations.

#### Genes

Thirteen gene-related prediction cases were used to predict attempt; each of these cases pertained to a serotonin-related gene: serotonin synthesis (polymorphisms spanning the tryptophan hydroxylase gene) and polymorphisms along serotonin transporter genes (5-HTT, 5-HTTLPR). The effect on suicide attempts was not significant, with a weighted mean OR of 1.30 (0.90–1.88). Four cases included suicide death as an outcome, generating a nonsignificant weighted mean OR of 0.73 (0.43–1.23).

#### Hormones

A total of three prediction cases used hormones to predict suicide attempt; no cases included suicide death as an outcome. The weighted mean effect of hormones on suicide attempt was not significant (OR=2.08 (0.66–6.57)).

#### Molecule binding

A total of four prediction cases were categorized as factors related to molecule binding. All cases were HRs drawn from a single study and used to predict suicide attempt. The weighted mean HR was 1.01 (0.99–1.03) and not significant.

#### Nutrients

Lewis *et al.* (2012) produced many suicide death prediction cases related to lowered levels of fish oil nutrients, but to reduce case dependence, we only included the strongest prediction case from each general type of nutrient (*n*=4; that is, Omega-3, Omega-6, monosaturated fatty acid and saturated fatty acid). These cases produced a weighted OR of 1.12 (1.02–1.22). Publication bias analyses indicated that one case below the mean was missing, and estimated an adjusted weighted mean OR of 1.09 (1.01–1.19).

#### Peripheral physiology

Only one prediction case was available (that is, blood pressure for suicide attempt), precluding a meta-analysis.

### Protective factor category analyses

Only three studies (four samples) included predictors that were categorized as protective factors. Only one of these studies included OR statistics, precluding a meta-analysis.^16^ The remaining two studies produced a nonsignificant weighted HR of 0.87 (0.66–1.15).^15, 17^

### Moderation analyses

#### Sample type

Prediction of suicide attempt was similar across general (*n*=8; weighted mean OR=1.56 (1.30–1.87)), clinical (*n*=13; weighted mean OR=1.36 (0.61–3.04)) and suicidal samples (*n*=14; weighted mean OR=1.50 (1.09–2.09)). Predicting suicide death, the strongest effects were detected among clinical samples (*n*=13; weighted mean OR=2.00 (1.41–2.84)), with weaker effects in general (*n*=16; weighted mean OR=1.14 (0.99–1.31)) and suicidal samples (*n*=13; weighted mean OR=1.39 (1.04–1.86)). There were too few cases to conduct similar analyses for HR cases and protective factors.

#### Length of follow-up

Meta-regression analyses did not indicate a significant effect of follow-up length on the magnitude of effect estimates predicting suicide attempt (*B*=−0.003 (−0.009 to 0.0032)) or suicide death (*B*=0.0012 (−0.0013 to 0.0038)). There were too few HR and protective factor cases to conduct similar analyses.

## Discussion

Suicidal behavior is a major public health problem that has not decreased appreciably in recent decades. In an effort to help improve the prediction and potential prevention of suicidal behavior, this meta-analysis tested whether any biological factors showed promise as potential risk factors for suicide attempt or death by suicide. Unfortunately, analyses revealed that the biological factors tested had a weak and, in most cases, nonsignificant association with subsequent suicide attempt or death. Only two specific biological factors remained significant after controlling for publication bias (that is, cytokines and low levels of fish oil nutrients), and there was only one study examining each of these factors. There also was no evidence of protective effects for any of the biological factors examined, and there was only one relevant case that included suicide ideation as an outcome. Whereas studies have shown that several biological factors correlate with suicidal behaviors,^6, 7, 8^ relatively few studies have tested whether these correlates prospectively predict suicidal behaviors (that is, are risk factors). Moreover, these studies typically included few participants with a suicide-related outcome, reducing the reliability of findings and increasing likelihood of publication bias. Indeed, results revealed that publication bias was high across all risk-factor categories and outcomes. These findings highlight a need for more biological risk factor studies, especially large studies with a large number of participants with a suicide-related outcome.

Meta-analytic results revealed that overall prediction of suicide attempt (weighted mean odds ratio (wOR)=1.41) and suicide death (wOR=1.23) were similar, but neither effect remained significant after accounting for publication bias. These were uniform findings as further analyses indicated that risk factor magnitude was not moderated by sample type or follow-up length. Results for specific risk-factor categories were similar, with only one biological factor subtype reaching significance after accounting for publication bias (and few reaching significance before accounting for publication bias). Although the present meta-analysis may have had limited power to detect small effects, it is notable that all effects—significant and nonsignificant—were uniformly small (that is, near or below 2.0). The results of our study differ from an earlier review and meta-analyses looking at potential biofactors which found that low CSF 5 H-IAA and HPA axis dysfunction were associated with odd ratios of suicide for 4.48 and 4.65, respectively.^41, 42^ We believe our conclusions different from this earlier meta-analysis for a number of reasons including our ability to include several more recent relevant studies published after the initial study^10, 37^ as well as the inclusion of other studies that met our inclusion criteria that had reported relatively weak findings.^36^ Given the low annual base rates of suicide attempts (0.32 per 100 people) and suicide death (0.013 per 100 people) in the United States,^2, 4^ this suggests that biological factors may not substantially increase the risk of future suicidal behaviors. For example, in our meta-analysis, CSF serotonin metabolite analyses produced a weighted OR of 2.15 for suicide death (before accounting for publication bias). In terms of absolute risk for suicide death for a given individual over a 1-year period, low CSF serotonin metabolites would increase risk from 0.013 per 100 people to 0.028 per 100 people, providing limited improvement in clinical prediction.

The present findings must be interpreted in the context of several important limitations. First, the failure to identify any strong associations between biological factors and suicide attempt or death may have been influenced by the design of existing studies. Nearly all studies included long follow-up intervals, measured factors as trait-like entities and tested putative risk factors in isolation. It is possible that, in the context of several other risk factors (for example, other biological factors, stressful life events, low social support, prior history of self-injury and hopelessness), sudden shifts in certain biological factors may be associated with greatly increased risk for a few hours, days or weeks. These findings highlight a need for studies that examine potential biological risk factors over very short intervals, in conjunction with other potential risk factors, and in a state-like manner.

Second, there were relatively few qualifying studies and most of these studies included few participants with a suicide-related outcome. Although our search for studies was broad, it is possible that the present meta-analysis did not include all relevant studies. The results showed uniformly small-risk-factor magnitudes across specific biological categories, outcomes, sample types and follow-up lengths, suggesting that a large number of additional studies would have produced similar findings. Nevertheless, more studies are needed in this area—particularly large studies that can generate reliable effect estimates.

Third, we did not include unpublished studies. On balance, as these unpublished studies are most likely to have obtained null or negative findings, this meta-analysis likely provided an optimistic assessment of the magnitude of biological risk factors for suicidal behaviors. Although we employed techniques to account for this publication bias, it is difficult to accurately estimate this bias within such a small literature.

The results suggest that few biological risk factors for suicidal behaviors have been identified. These findings present a sobering view of our current knowledge of biological risk factors for suicide. We hope that recognition of the current state of knowledge will serve as an impetus for future projects and directions. Important future directions in the investigation of novel biological factors include factors related to brain imaging, psychophysiology (for example, heart rate variables, electroencephalography and heart rate variability), changes in gene expression, and possible factors associated with changes in gene expression (for example, miRNA and metabolic processes). It may also be helpful to investigate biological aspects of psychological risk factors for suicidal behavior. For example, measuring physiological reactions to suicide-related stimuli may provide an effective measure of suicidal capability. Other future work focused on studies with shorter time intervals, interactions between biological and environmental factors, and changes in biological factors over short periods of time may build on past work and provide key insights into suicide risk detection and prevention.^43^

## Figures and Tables

**Table 1:** Biological factors studied

Blood-related factors: glucose; cholesterol; serum tryptophan ratio; plasma oxytocin
Cerebrospinal fluid metabolite: serotonin; dopamine; oxytocin; norepinephrine; cortisol-releasing hormone; dehydroepiandrosterone
Cytokines: monocyte chemotactic protein-1; tumor necrosis factor-a; vascular endothelial growth factor; interleukin-10
Genes: serotonin synthesis (tryptophan hydroxylase gene); serotonin transporter and receptor poloymorphisms (5-HTT, 5-HTTLPR)
Hormone challenges/tests: nonsuppression (or lowered suppression) on the dexamethasone suppression test; fenfluramine challenge
Molecule binding: affinity constant of platelet serotonin [3H] paroxetine binding; maximum number of binding site (Bmax) of [3H] paroxetine
Nutrients: serum cholesterol level; omega-3; omega-6; monosaturated fatty acid; saturated fatty acid intake and serum levels
Peripheral physiology: systolic blood pressure; forced vital capacity
